# Are there multiple ways to direct attention in working memory?

**DOI:** 10.1111/nyas.13634

**Published:** 2018-04-10

**Authors:** Amy L. Atkinson, Ed D.J. Berry, Amanda H. Waterman, Alan D. Baddeley, Graham J. Hitch, Richard J. Allen

**Affiliations:** ^1^ School of Psychology University of Leeds Leeds United Kingdom; ^2^ Department of Psychology University of York Heslington York United Kingdom

**Keywords:** visual working memory, attention, focus of attention, probe frequency, probe value, prioritization

## Abstract

In visual working memory tasks, memory for an item is enhanced if participants are told that the item is relatively more valuable than others presented within the same trial. Experiment 1 explored whether these probe value boosts (termed *prioritization effects* in previous literature) are affected by probe frequency (i.e., how often the more valuable item is tested). Participants were presented with four colored shapes sequentially and asked to recall the color of one probed item following a delay. They were informed that the first item was more valuable (differential probe value) or as valuable as the other items (equal probe value), and that this item would be tested more frequently (differential probe frequency) or as frequently (equal probe frequency) as the other items. Probe value and probe frequency boosts were observed at the first position, though both were accompanied by costs to other items. Probe value and probe frequency boosts were additive, suggesting the manipulations yield independent effects. Further supporting this, experiment 2 revealed that probe frequency boosts are not reliant on executive resources, directly contrasting with previous findings regarding probe value. Taken together, these outcomes suggest there may be several ways in which attention can be directed in working memory.

## Are there multiple ways to direct attention in working memory?

Working memory (WM) refers to a system that allows a limited amount of information to be temporarily stored in a state of heightened accessibility for use in ongoing information processing.^1^ It is considered essential for a myriad of important activities, including learning and skill acquisition.^2^, ^3^ Many of these activities require individuals to retain information that varies by importance or goal revelance.^4^, ^5^ An attentional mechanism that allows a subset of information to be stored in a privileged state would therefore be highly advantageous.^6^ There is growing evidence to suggest that such a subregion exists within WM, termed the *focus of attention* (FoA).^7^, ^8^, ^9^, ^10^

The FoA, and the relationship between WM and attention more generally, has been explored using several methods. The most commonly employed paradigm is retro‐cueing, in which a cue is presented immediately following an array of to‐be‐remembered items. This cue typically informs participants which item will, or is most likely to, be tested at retrieval. Retro‐cues improve memory for the cued item,^11^, ^12^, ^13^, ^14^ though the size of boosts appear to depend on the reliability of the cue.^12^ For instance, Gunseli *et al*.^12^ reported larger benefits when the cued item was tested 80% of the time, compared with a condition in which the cued item was assessed in only 50% of the trials.

The reward associated with an item can also be increased to encourage participants to direct attention toward it.^15^, ^16^, ^17^, ^18^, ^19^, ^20^, ^21^, ^22^ In recent years, this has been used to explore the relationship between WM and attention through a probe value manipulation (often referred to as “strategic prioritization” in previous literature^15^, ^16^, ^17^, ^18^). In this paradigm, participants are typically presented with a series of colored shapes sequentially. After a brief delay, memory for one of the shape‐color bindings is assessed using cued recall. Before the block of trials, participants are told that one serial position (SP) is more valuable than the rest, with correct recall of that item gaining them more points. For example, participants might be told that correct recall of the first SP will gain them four points, whereas correct recall of any other item will gain them one point.^16^ Although the points system is notional, this results in a memory boost for the more valuable item relative to a condition in which the same SP is less valuable (i.e., worth fewer points). This boost is, however, accompanied by costs to other items presented within the same trial, which are not remembered as accurately.^15^, ^16^, ^17^ Regardless of which SP is more valuable, participants also show a robust recency effect, exhibiting higher accuracy at the final position relative to other nonprioritized positions.^15^ From this, it has been concluded that the more valuable item and the final item are more likely to be retained in the FoA, rendering them more accessible.^15^, ^16^, ^17^ Boosts to the more valuable item (referred to as probe value boosts hereafter) are reduced by an attentionally demanding concurrent task, suggesting that these effects are likely to involve a process that relies on executive control, such as attentional refreshing.^16^ In contrast, the recency effect is not reduced by a concurrent task, suggesting that items can be retained in the FoA through cost‐free automatic routes, as well as costly voluntary routes.^16^

In research using the probe value paradigm, memory for the more valuable item has been tested as frequently as memory for the other items.^15^, ^16^, ^17^ However, evidence from the cueing literature suggests that the size of retro‐cue boosts depend on the frequency with which the cued item is assessed at retrieval.^12^ The size of probe value boosts might therefore also differ depending upon how often the more valuable item is tested (i.e., probe frequency). Such findings would provide further insights into the probe value effect, demonstrating whether boosts are affected by other task factors. These findings would also reveal whether probe value and probe frequency effects are independent or contingent on each other. Evidence that probe value and probe frequency effects are independent might suggest that the manipulations encourage participants to direct attention in different ways, a finding that would have important implications for the relationship between WM and attention.

These research questions were investigated in the current set of experiments. Experiment 1 orthogonally manipulated probe value and probe frequency, targeting both manipulations at the first item. Experiment 2 explored probe frequency effects further, examining whether boosts are reliant on executive resources during encoding and maintenance, as probe value effects appear to be.^16^

## Experiment 1

Experiment 1 orthogonally manipulated probe value (whether an item was more valuable (differential) or not (equal)) and probe frequency (whether an item was more likely to be tested (differential) or not (equal)). Both manipulations were targeted at the first item. Based on previous findings, it was predicted that significant probe value boosts would be observed at the first SP, whereby memory for this item would be higher in the differential probe value condition than the equal probe value condition.^15^, ^16^ It was also predicted that probe frequency effects would emerge at SP1, with participants exhibiting higher accuracy for this item in the differential probe frequency condition than the equal probe frequency condition.^11^, ^12^, ^13^ Of particular interest was whether an interaction would emerge at SP1. Evidence of an interaction between probe value and probe frequency would indicate that these manipulations are not independent, and that the size of probe value boosts differs depending on probe frequency. Moreover, evidence that probe value effects are smaller in the differential probe frequency condition would suggest that participants experience less benefit from increased probe value when they are already motivated to direct attention to this item. Such findings might be taken as evidence that probe value and probe frequency encourage participants to direct attention in similar ways. Alternatively, probe value and probe frequency effects might be additive, suggesting that the manipulations might encourage participants to direct attention in different ways.

### Method

#### Participants

Forty‐four young adults took part (aged 18–30 years; *M* = 20.42; SD = 1.15; nine males). Participants were native English speakers with no known learning difficulties, normal or corrected‐to‐normal vision, and no color blindness. Participants were undergraduate students who were reimbursed for their time with course credits. The experiment was approved by the School of Psychology Ethics Committee at the University of Leeds (UK).

#### Materials

In each trial, four items were presented sequentially. Stimuli were created by randomly pairing a shape from a pool of six options (circle, triangle, arch, arrow, flag, cross) with a color from a pool of six options (red, yellow, green, blue, purple, black). No shape or color was repeated within the same trial. All stimuli subtended a visual angle of approximately 1.5°, based on a viewing distance of 50 cm. Shapes were presented on a white background at one of eight points around a 2° imaginary circle positioned at the center of the screen. Locations were selected randomly, with the constraint that no location could be used more than once per trial. The test cue was an outline of one of the stimuli presented during the encoding phase. This was displayed in the center of the screen. In the equal probe frequency condition, the first SP was tested as often as other items (25% of the time). In the differential probe frequency condition, the first SP was assessed 70% of the time, whereas the other items were each probed 10% of the time.

#### Design and procedure

The study employed a 2 × 2 × 4 within subject design, manipulating probe value (differential, equal), probe frequency (differential, equal), and SP (1–4). Participants completed four blocks of 40 trials; one for each combination of probe value and probe frequency conditions. The order of probe frequency blocks and the order of probe value blocks within the probe frequency conditions were counterbalanced. In the equal probe frequency conditions, each SP was tested 10 times. In the differential probe frequency conditions, the first SP was tested 28 times and the other SPs were each tested four times. The SPs tested were randomly distributed within the blocks. At the start of each block, participants completed four practice trials to familiarize themselves with the condition.

Each condition commenced with the provision of written instructions. In the differential probe value conditions, participants were told that correct recall of the first item would earn them four points, whereas correct recall of the other items would earn them one point. In the equal probe value conditions, they were told each item was worth one point. The points were part of a notional reward system: the number of points accrued was never tallied and no actual rewards were given. During the instructions, participants were also informed about the probe frequency manipulation. In the equal probe frequency conditions, they were told that all items would be tested the same number of times. In the differential probe frequency condition, they were told that the first item would be tested more often than the other items.

**Figure 1:**
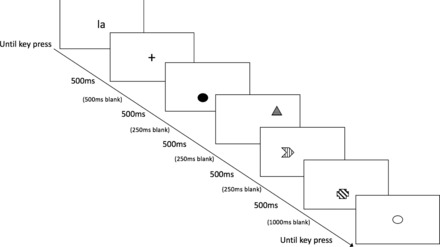
The experimental paradigm used in experiment 1.

The experimental paradigm used is displayed in Figure 1. Each trial began with presentation of the word “la,” which participants were asked to whisper until the retrieval phase to prevent verbal recoding.^23^ Following a key press, a fixation cross appeared for 500 ms, which informed participants that the shapes were about to appear. Next, four colored shapes were displayed sequentially, with each shape displayed for 500 milliseconds. After a delay of 1000 ms, the outline of one shape was presented and participants verbally recalled the original color of the shape. Their response was recorded by the experimenter, who then pressed the space bar to progress onto the next trial. Participants were reminded of the probe value and probe frequency instructions after every ten trials. Participants were not given feedback regarding performance on the task.

### Data analysis

The dependent variable was accuracy, determined by the proportion of trials where participants responded correctly. Findings are first reported as a function of SP. Further planned analysis was then conducted at SP1, as this is the position at which the manipulations were targeted.

In experiment 1 and the subsequent experiments, data were analyzed using frequentist and Bayes Factor (BF) methods. BF analysis assesses the strength of evidence for the alternative hypothesis relative to the null hypothesis, and also provides a test of equivalence between conditions.^24^, ^25^ This analysis was conducted in R,^26^ using the BayesFactor package.^27^ When reporting the BF analysis, the most likely model given the data is described relative to the null model including only random effects of participant. BFs for all main effects and interactions are also reported. If the effect or interaction was included in the most likely model, the BF was calculated by comparing the most likely model to a model excluding that effect. If the effect or interaction was not included in the most likely model, the BF was calculated by comparing the most likely model to a model including all of the effects featured in the most likely model plus the effect of interest. BF_10_ values describe how many times more likely the alternative hypothesis is to the null hypothesis, whereas BF_01_ describes the ratio of how likely the null hypothesis is compared with the alternative hypothesis.

### Results

#### Across SPs

**Figure 2:**
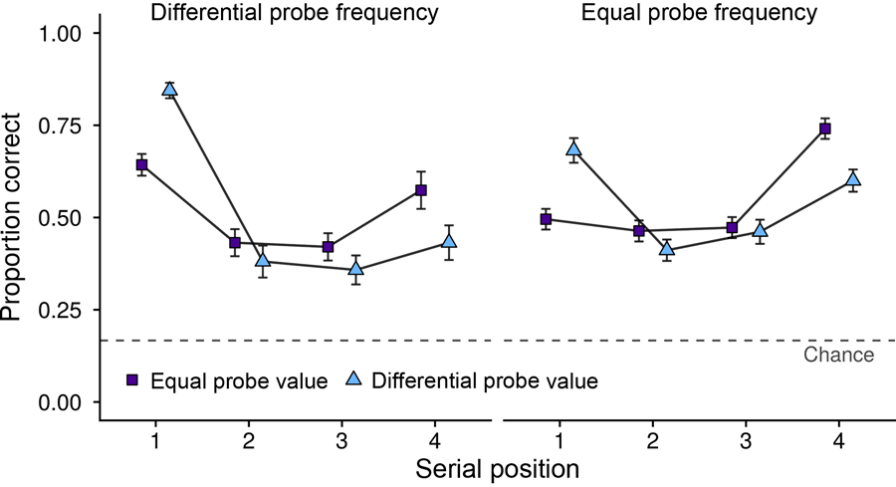
Mean accuracy (and SE) as a function of probe value, SP, and probe frequency in experiment 1.

Mean accuracy as a function of probe value, probe frequency, and SP is displayed in Figure 2. A 2 (Probe value) × 2 (Probe frequency) × 4 (SP) within‐subjects analysis of variance (ANOVA) revealed no main effect of probe value (Differential *M* = 0.52, SE = 0.02; Equal M = 0.53, SE = 0.02; (*F*(1,43) = 0.38, *P* = 0.54, MSE = 0.038, $\eta_{p}^{2}$ < 0.01, $\eta_{G}^{2}$ < 0.01; BF_01_ = 10.09) or probe frequency (Differential *M* = 0.51, SE = 0.02; Equal M = 0.54, SE = 0.02; *F*(1,43) = 2.57, *P* = 0.12, MSE = 0.063, $\eta_{p}^{2}$ = 0.056, $\eta_{G}^{2}$ < 0.01; BF_01_ = 2.01), demonstrating that neither manipulation affected overall performance on the task. A main effect of SP emerged (*F*(3,129) = 40.07, *P* < 0.001, MSE = 0.064, $\eta_{p}^{2}$ = 0.48, $\eta_{G}^{2}$ = 0.17; BF_10_ > 1000). Pairwise comparisons (corrected using Bonferroni–Holm) revealed significant differences between SP1 (*M* = 0.66, SE = 0.02) and SP2 (*M* = 0.42, SE = 0.02; *P* < 0.001), SP1 and SP3 (*M* = 0.43, SE = 0.02; *P* < 0.001), SP1 and SP4 (*M* = 0.59, SE = 0.03; *P* = 0.034), SP2 and SP4 (*P* < 0.001), and SP3 and SP4 (*P* < 0.001). Significant interactions emerged between probe value and SP (*F*(3,129) = 25.01, *P* < 0.001, MSE = 0.036, $\eta_{p}^{2}$ = 0.37, $\eta_{G}^{2}$ = 0.069; BF_10_ > 1000), and probe frequency and SP (Greenhouse‐Geisser corrected *F*(2.54,109.31) = 19.15, *P* < 0.001, MSE = 0.042, $\eta_{p}^{2}$ = 0.31, $\eta_{G}^{2}$ = 0.063; BF_10_ > 1000), indicating that the effects of probe value and probe frequency differed depending upon the SP tested. No interactions emerged between probe value and probe frequency (*F*(1,43) = 0.11, *P* = 0.74, MSE = 0.032, $\eta_{p}^{2}$ < 0.01, $\eta_{G}^{2}$ < 0.01; BF_01_ = 8.34) or probe value, probe frequency, and SP (Greenhouse–Geisser corrected *F*(2.51,107.82) = 0.25, *P* = 0.83, MSE = 0.037, $\eta_{p}^{2}$ < 0.01, $\eta_{G}^{2}$ < 0.01; BF_01_ = 27.22). These outcomes were corroborated by BF analysis, which revealed that the most likely model included a main effect of SP, as well as interactions between probe value and SP, and probe frequency and SP (BF_10_ > 1000 relative to the null model with random effects of participant only).

To investigate the interaction between probe value and SP, a series of paired sample *t*‐tests (corrected using Bonferroni–Holm) were conducted. Mean proportion correct as a function of probe value and SP is displayed in Table 1. At SP1, participants performed significantly better in the differential probe value condition (*t*(43) = 8.85, *P* < 0.001, BF_10_ > 1000, *d* = 1.33). The pattern was reversed at SP4, with participants exhibiting higher accuracy in the equal probe value condition (*t*(43) = –4.08, *P* < 0.001, BF_10_ = 130.6, *d* = –0.61). No significant differences between probe value conditions were found at SP2 (*t*(43) = –1.71, *P* = 0.19, BF_01_ = 1.64, *d* = –0.26) or SP3 (*t*(43) = –1.38, *P* = 0.19, BF_01_ = 2.56, *d* = –0.21). In summary, this indicates that increasing the value of the first item boosted performance at SP1, had no significant effect on performance at SP2 and SP3, and negatively affected performance at SP4.

**Table 1:** Mean accuracy (and SE) in experiment 1 as a function of probe value and SP, collapsed across probe frequency conditions

	SP1	SP2	SP3	SP4	Across SPs
Differential probe value	0.76 (0.02)	0.40 (0.03)	0.41 (0.03)	0.52 (0.03)	0.52 (0.02)
Equal probe value	0.57 (0.02)	0.45 (0.02)	0.45 (0.03)	0.66 (0.03)	0.53 (0.02)

A series of paired sample *t*‐tests (corrected using Bonferroni–Holm) were also conducted to investigate the interaction between probe frequency and SP. Mean proportion correct as a function of probe frequency and SP is displayed in Table 2. At SP1, higher accuracy was observed in the differential probe frequency condition (*t*(43) = 6.05, *P* < 0.001, BF_10_ > 1000, *d* = 0.91). The opposite pattern of results was observed at SP3 and SP4, with participants exhibiting significantly higher accuracy in the equal probe frequency condition (SP3: *t*(43) = –2.47, *P* = 0.035, BF_10_ = 2.45, *d* = –0.37; SP4: *t*(43) = –4.51, *P* < 0.001, BF_10_ = 450.47, *d* = –0.68). No effect of probe frequency emerged at SP2 (*t*(43) = –0.87, *P* = 0.39, BF_01_ = 4.35, *d* = –0.13). To summarize, this demonstrates that increasing the likelihood of the first item being assessed enhanced accuracy at SP1, had no significant effect at SP2, and reduced accuracy at SP3 and SP4.

**Table 2:** Mean accuracy (and SE) in experiment 1 as a function of probe frequency and SP, collapsed across probe value conditions

	SP1	SP2	SP3	SP4	Across SPs
Differential probe frequency	0.74 (0.02)	0.41 (0.03)	0.39 (0.03)	0.50 (0.04)	0.51 (0.02)
Equal probe frequency	0.59 (0.03)	0.44 (0.02)	0.47 (0.03)	0.67 (0.03)	0.54 (0.02)

#### SP1

As both manipulations were targeted at SP1, further analysis was conducted at this SP to explore whether an interaction emerged between probe value and probe frequency. A 2 (Probe value) × 2 (Probe frequency) within‐subjects ANOVA revealed a significant main effect of probe value (*F*(1,43) = 78.28, *P* < 0.001, MSE = 0.021, $\eta_{p}^{2}$ = 0.65, $\eta_{G}^{2}$ = 0.21; BF_10_ > 1000), with participants exhibiting higher accuracy in the differential probe value condition. There was also a significant main effect of probe frequency (*F*(1,43) = 36.57, *P* < 0.001, MSE = 0.029, $\eta_{p}^{2}$ = 0.46, $\eta_{G}^{2}$ = 0.15; BF_10_ > 1000), with participants exhibiting higher accuracy in the differential probe frequency condition. No significant interaction emerged between probe value and probe frequency (*F*(1,43) = 0.17, *P* = 0.69, MSE = 0.015, $\eta_{p}^{2}$ < 0.01, $\eta_{G}^{2}$ < 0.01; BF_01_ = 4.22), suggesting that probe value boosts do not differ depending on probe frequency and that the manipulations have independent effects. BF analysis supported these conclusions, with the most likely model including main effects of probe value and probe frequency (BF_10_ > 1000 relative to the null model with random effects of participant only).

### Discussion

In line with previous findings, participants exhibited significant probe value effects, providing further evidence that individuals can direct attention to more valuable items in visual WM.^15^, ^16^, ^17^ Significant probe frequency effects were also observed, demonstrating that individuals can also orient attention to items that are more likely to be tested. Both probe value and probe frequency boosts came at a cost to some of the other items, suggesting that these manipulations do not increase WM capacity, but rather encourage individuals to alter the way in which they allocate attention. Evidence that costs emerged in the probe frequency condition should, however, be treated with caution, as there were only a small number of trials testing SPs 2–4 in the differential probe frequency conditions. Nevertheless, this is in line with previous findings, suggesting that the direction of attention within WM results in costs to items that are not focused on.^12^, ^13^, ^15^, ^16^, ^17^

Importantly, no significant interaction emerged between probe value and probe frequency across SPs or at the SP in which the manipulations were targeted (SP1). This indicates that probe value effects are not affected by the frequency with which the more valuable item is assessed at retrieval. Perhaps more importantly, this indicates that probe value and probe frequency are independent in their impacts on WM, and that they might encourage individuals to direct attention in different ways. Evidence for the latter is preliminary, however, warranting additional research to explore this possibility further.

Previous research has suggested that probe value effects are reduced if participants engage in an attentionally demanding concurrent task during encoding and maintenance.^16^ It would be useful to establish whether such a task also reduces probe frequency boosts. This would provide further insights into probe frequency effects, while also further exploring whether probe value and probe frequency are likely to encourage participants to direct attention in different ways. Experiment 2 therefore investigated this.

## Experiment 2

Hu *et al*.^16^ recently explored whether probe value effects are reliant on executive resources. In their experiment, participants were told that the first SP or the final SP was more valuable than the rest. During encoding and maintenance, participants either engaged in articulatory suppression (low load) or counted upwards in steps of two (high load). Significant probe value boosts were observed in the low load condition, though these were significantly reduced or abolished under high load. From this, it was concluded that probe value effects are reliant on executive control.^16^

To the best of our knowledge, research to date has not explored whether probe frequency boosts in WM also rely on executive control. However, a series of studies have demonstrated that individuals reliably encode frequency information during memory tasks^28^ (but see Ref. 29 for a review). The accuracy of these frequency judgments appears to be unaffected by age, intentionality, feedback, or practice,^30^, ^31^ suggesting that frequency information might be encoded automatically.^28^, ^32^ Furthermore, evidence from amnesic patients suggests that this group can use recurring patterns to enhance performance on motor tasks, despite not being explicitly aware that a pattern is being repeated.^33^ This suggests that individuals may also apply frequency information automatically. As such, probe frequency boosts in WM might occur in a relatively cost‐free manner, placing minimal reliance on executive resources. Such findings would provide evidence of a dissociation between probe value and probe frequency, providing further evidence that the manipulations encourage participants to direct attention in different ways. This was therefore explored in Experiment 2.

To allow comparisons with the probe value literature, the methodology used was similar to that used by Hu *et al*.^16^ A 2 (probe frequency: equal, differential) × 2 (load: equal, differential) × 4 (SP: 1–4) within‐subjects design was employed. In the low load condition, participants simply repeated a two‐digit number during encoding and maintenance. In the high load conditions, participants counted upwards in steps of two during these phases. As in experiment 1, the probe frequency manipulation was targeted at the first SP. Performance at this position was therefore of particular interest. Evidence of an interaction between probe frequency and load, with performance in the differential probe frequency condition particularly affected by an increase in load, would suggest that boosts are reliant on executive control. These outcomes would be in line with findings from the probe value literature.^16^ Conversely, evidence of no interaction between probe frequency and load would suggest that effects are not reliant on executive resources, contrasting with findings on probe value.^16^ Such outcomes would suggest that the manipulations encourage participants to direct attention in different ways, further supporting the conclusions drawn from experiment 1.

### Method

#### Participants

Twenty‐four young adults participated (aged 18–35 years; *M* = 22.11, SD = 3.58; 10 males). Participants were either paid or given course credit. The experiment was approved by the School of Psychology Ethics Committee at the University of Leeds (UK).

#### Materials

The materials used were identical to experiment 1, except that participants were presented with a randomly selected number between 20 and 99 at the start of each trial, as opposed to the word “la.”

#### Design and procedure

The study employed a 2 × 2 × 4 within‐subjects design, manipulating probe frequency (equal, differential), load (low, high) and SP (1–4). Participants completed four blocks of 40 trials; one for each combination of probe frequency and load. The order of probe frequency blocks and the order of load blocks within the probe frequency conditions were counterbalanced. The SPs tested were randomly distributed within the blocks.

Participants were told that correctly recalling the color of the shape tested would gain them one point. The probe frequency instructions were the same as in experiment 1. The experimental paradigm was also identical to experiment 1, except that participants were presented with a number between 20 and 99 at the start of the trial as opposed to the word “la.” In the low load conditions, participants were asked to repeat the number until retrieval. In the high load conditions, participants were asked to count upwards in steps of two from the number until the retrieval phase (e.g., 45, 47, 49). This load manipulation is identical to that employed by Hu *et al*.

### Data analysis

The dependent variable was accuracy, determined by mean proportion correct. Findings are first reported across SPs, followed by further planned analysis at SP1.

### Results

#### Across SPs

**Figure 3:**
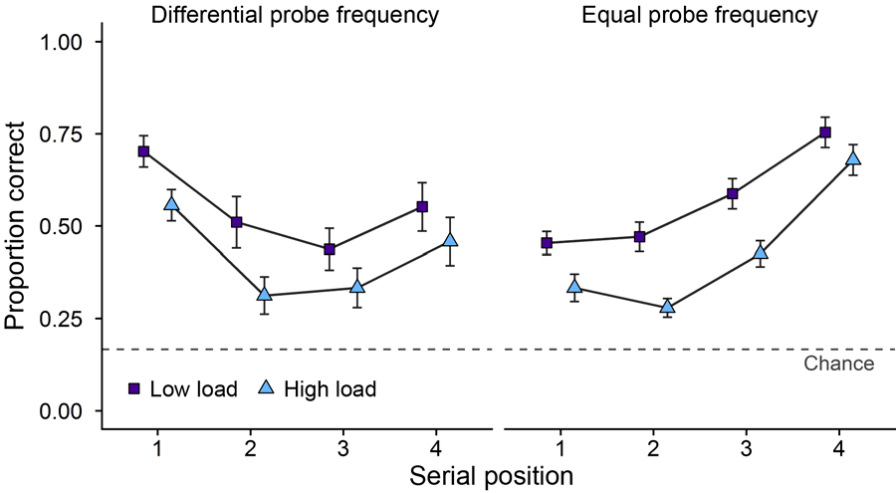
Mean proportion correct (and SE) as a function of probe frequency, SP, and load in experiment 2.

Mean proportion correct as a function of probe frequency, SP, and load is displayed in Figure 3. A 2 (Probe frequency) × 2 (Load) × 4 (SP) within‐subjects ANOVA revealed no significant main effect of probe frequency (Differential *M* = 0.48, SE = 0.03; Equal M = 0.50, SE = 0.02; *F*(1,23) = 0.39, *P* = 0.54, MSE = 0.056, $\eta_{p}^{2}$ = 0.016, $\eta_{G}^{2}$ < 0.01; BF_01_ = 7.25), indicating that increasing the likelihood of the first item being assessed did not affect overall performance on the task. There was, however, a main effect of load (*F*(1,23) = 77.86, *P* < 0.001, MSE = 0.023, $\eta_{p}^{2}$ = 0.77, $\eta_{G}^{2}$ = 0.081; BF_10_ > 1000), with higher accuracy in the low load condition (*M* = 0.56, SE = 0.02) relative to the high load condition (*M* = 0.42, SE = 0.21). There was also a significant main effect of SP (Greenhouse–Geisser corrected *F*(2.19,50.39) = 10.48, *P* < 0.001, MSE = 0.081, $\eta_{p}^{2}$ = 0.31, $\eta_{G}^{2}$ = 0.11; BF_10_ > 1000), with pairwise comparisons (corrected using Bonferroni–Holm) revealing significant differences at SP1 (*M* = 0.51, SE = 0.03) and SP2 (*M* = 0.39, SE = 0.03; *P* = 0.012), SP2 and SP4 (*M* = 0.61, SE = 0.04; *P* < 0.001) and SP3 (*M* = 0.45, SE = 0.03) and SP4 (*P* < 0.001). A significant interaction emerged between probe frequency and SP (*F*(3,69) = 17.79, *P* < 0.001, MSE = 0.052, $\eta_{p}^{2}$ = 0.44, $\eta_{G}^{2}$ = 0.12; BF_10_ > 1000), indicating that the effects of probe frequency differed depending on the SP tested. In contrast, there was no interaction between load and SP (*F*(3,69) = 1.31, *P* = 0.28, MSE = 0.037, $\eta_{p}^{2}$ = 0.054, $\eta_{G}^{2}$ < 0.01; BF_01_ = 10.96). There were also no interactions between probe frequency and load (*F*(1,23) < 0.01, *P* = 0.96, MSE = 0.034, $\eta_{p}^{2}$ < 0.01, $\eta_{G}^{2}$ < 0.01; BF_01_ = 6.51), and probe frequency, load and SP (*F*(3,69) = 0.27, *P* = 0.85, MSE = 0.033, $\eta_{p}^{2}$ = 0.012, $\eta_{G}^{2}$ < 0.01; BF_01_ = 14.2). These findings were corroborated by BF analysis, which revealed that the most likely model included main effects of load and SP, as well as an interaction between probe frequency and SP (BF_10_ > 1000 relative to the null model with random effects of participant only).

To investigate the interaction between probe frequency and SP, a series of *t*‐tests (corrected using Bonferroni–Holm) were conducted. Accuracy as a function of probe frequency and SP is displayed in Table 3. Accuracy was higher in the differential probe frequency condition at SP1 (*t*(23) = 6.63, *P* < 0.001, BF_10_ > 1000, *d* = 1.35). This pattern was reversed at SP3 (*t*(23) = –2.78, *P* = 0.021, BF_10_ = 4.63, *d* = –0.57) and SP4 (*t*(23) = –3.65, *P* = 0.004, BF_10_ = 27.09, *d* = –0.74), with participants exhibiting higher accuracy in the equal probe frequency condition. No significant effect of probe frequency emerged at SP2 (*t*(23) = 0.76, *P* = 0.46, BF_01_ = 3.57, *d* = 0.15). To summarize, these outcomes indicate that increasing the likelihood of the first item being tested enhanced memory at SP1, had no significant effect at SP2, and impaired memory at SP3 and SP4.

**Table 3:** Mean accuracy (and SE) in experiment 2 as a function of probe frequency and SP, collapsed across load conditions

	SP1	SP2	SP3	SP4	Across SPs
Differential probe frequency	0.63 (0.04)	0.41 (0.05)	0.39 (0.05)	0.51 (0.06)	0.48 (0.03)
Equal probe frequency	0.39 (0.02)	0.38 (0.03)	0.51 (0.03)	0.72 (0.04)	0.50 (0.02)

#### SP1

As in experiment 1, further planned analysis was conducted at SP1 to explore whether an interaction emerged between probe frequency and load. A 2 (Probe frequency) × 2 (Load) within‐subjects ANOVA revealed a main effect of probe frequency (*F*(1,23) = 43.89, *P* < 0.001, MSE = 0.03, $\eta_{p}^{2}$ = 0.66, $\eta_{G}^{2}$ = 0.29; BF_10_ > 1000), with participants exhibiting higher accuracy in the differential probe frequency condition relative to the equal probe frequency condition. There was also a main effect of load (*F*(1,23) = 18.66, *P* < 0.001, MSE = 0.023, $\eta_{p}^{2}$ = 0.45, $\eta_{G}^{2}$ = 0.12; BF_10_ = 435.73), with participant exhibiting higher accuracy in the low load condition. There was, however, no significant interaction between probe frequency and load (*F*(1,23) = 0.24, *P* = 0.63, MSE = 0.016, $\eta_{p}^{2}$ = 0.01, $\eta_{G}^{2}$ < 0.01; BF_01_ = 3.33), suggesting that the probe frequency boosts observed were not affected by load. BF analysis revealed that the most likely model included main effects of probe frequency and load (BF_10_ > 1000 relative to the null model with random effects of participant only).

### Discussion

Replicating experiment 1, significant probe frequency effects were observed, providing further evidence that individuals can orient attention to items that are more likely to be tested within a visual sequence. Importantly, however, there was no interaction between probe frequency and load at SP1, suggesting that probe frequency effects are not reduced by an attentionally demanding concurrent task.

These outcomes suggest that probe frequency boosts are not reliant on executive resources during encoding and maintenance, and might be obtained in a relatively cost‐free and automatic manner.^28^, ^29^, ^32^ This directly contrasts with research exploring probe value, which has revealed that these effects are significantly reduced or abolished under high cognitive load.^16^ This suggests that the motivation underpinning attentional direction is important in determining whether boosts are reliant on executive control. It also provides further evidence that the probe value and probe frequency manipulations encourage participants to direct attention in different ways.

Evidence that probe frequency boosts are not reliant on executive resources also contrasts with findings from the retro‐cue literature. In a pair of experiments, Janczyk and Berryhill^34^ revealed that retro‐cue effects are significantly reduced if participants engage in an attentionally demanding concurrent task during cue onset and encoding. These contrasting findings might indicate that reliance on executive control depends on the stage at which individuals are told which item is most likely to be tested (i.e., before or after item presentation). However, these differences in findings could also relate to other methodological factors. In the current experiment, the first SP was always more likely to be tested in the differential probe frequency conditions. However, in retro‐cue experiments, the item that is more likely to be probed changes on a trial‐by‐trial basis. It might therefore be impossible for participants to automate the direction of attention in the retro‐cue paradigm, as we believe may be occurring here.

Increased probe frequency came at a cost to some SPs that were less likely to be tested (SP3 and SP4). This replicates the outcomes from experiment 1, further demonstrating that the direction of attention can negatively affect items that are not focused on.^12^, ^13^, ^15^, ^16^, ^17^ Across conditions, accuracy at SP4 was significantly higher than the other SPs that were less likely to be assessed (SP2 and SP3), supporting previous findings that this item holds a privileged status within WM.^15^ As in experiment 1, these outcomes should, however, be interpreted with caution, as participants completed only a small number of trials at SPs 2–4 in the differential probe frequency conditions.

## General discussion

A pair of experiments explored how attention can be directed in visual WM. Of particular interest was whether probe value boosts are affected by the probability with which the more valuable item is tested at retrieval (i.e., probe frequency) or whether these manipulations yield independent effects. In experiment 1, probe value and probe frequency boosts were observed, although the two effects were additive. This demonstrates that probe value boosts are not affected by probe frequency and that the manipulations have independent effects on performance. This latter finding was further supported by experiment 2, which indicated that probe frequency boosts are not reliant on executive resources during encoding and maintenance, unlike probe value effects.^16^ Taken together, these findings suggest that probe value and probe frequency encourage participants to direct attention in different ways.

Such findings might be taken as evidence that the manipulations involve distinct underlying mechanisms. But how might the boosts emerge? Probe value effects are thought to reflect the more valuable item being retained in the FoA for longer periods of time or more frequently relative to less valuable items.^15^, ^16^ As probe value boosts appear to rely on executive resources during encoding and maintenance,^16^ effects are likely to result from a process that relies on executive control occurring during one or both of these stages. One possibility is that probe value biases attentional refreshing,^16^ a process that retains information by reactivating decaying memory traces.^35^, ^36^, ^37^, ^38^ The more valuable item might be refreshed more frequently or for longer periods of time, thus keeping the representation active. In contrast, probe frequency effects do not appear to rely on executive resources suggesting that boosts might occur in a cost‐free and relatively automatic manner.^28^, ^29^, ^32^ Effects are therefore unlikely to result from processes that rely on executive control, such as attentional refreshing.^39^, ^40^, ^41^ Although speculative, one possibility is the item that is most likely to be probed is automatically tagged as being more important. This might occur because participants are told this item will be tested more frequently, or because they become explicitly or implicitly aware of this throughout the block of trials. The WM system might then respond to this information, with more goal‐relevant items being held in the FoA automatically. Alternatively, probe frequency effects might not involve the FoA, and instead reflect a biasing at a different stage of WM. For instance, items that are tagged as being more goal relevant might be encoded with greater strength^42^ or prioritized for comparison with the probe at retrieval.^5^

Alternatively, the probe value and probe frequency manipulations may involve the same underlying mechanism. Experiment 1 revealed additive effects when probe value and probe frequency were employed together, which we interpret as indicating the operation of independent underlying mechanisms. However, these outcomes might be expected if the manipulations involve the same mechanism, but neither fully saturate it. Instead of involving distinct mechanisms, the probe value and probe frequency manipulations might therefore activate the same mechanism but in different ways. Experiment 2 would then indicate that activation of this mechanism is somewhat automatic when probe frequency is increased, but under more strategic control when probe value is manipulated. To delineate between these possibilities, it would be useful for additional research to further explore how the probe value and probe frequency manipulations differ and the cognitive mechanisms involved in both.

Regardless of the outcomes of this further research, the current findings have important implications for the relationship between WM and attention, suggesting that not all forms of attentional direction are functionally equivalent. Researchers should therefore avoid assumptions that different attentional manipulations encourage participants to direct attention in the same way, as this could result in inaccurate or erroneous conclusions. These findings may also have important practical implications, indicating that individuals can direct attention to more important information in WM if they have prior knowledge regarding value or goal relevance. This might be particularly useful for everyday tasks that rely on WM, such as learning,^2^ skill acquisition,^3^ and language comprehension.^43^ It is important to note that the orientation of attention does not increase WM capacity, however, and that this might negatively affect memory for other items held within the system (but see Ref. 44).

In summary, these experiments suggest that the manipulation of probe value and probe frequency encourages participants to direct attention in different ways. Although probe value effects appear to depend on executive resources during encoding and maintenance,^16^ probe frequency effects do not. Taken together, these findings suggest that the manipulations may involve distinct underlying mechanisms, or at least activate the same mechanism in different ways, though further research is needed to fully explore this possibility.

## Competing interests

The authors declare no competing interests.
